# Energy efficient partition allocation in mixed-criticality systems

**DOI:** 10.1371/journal.pone.0213333

**Published:** 2019-03-18

**Authors:** Ana Guasque, Patricia Balbastre, Alfons Crespo, Salva Peiró

**Affiliations:** 1 Institut d’Automàtica i Informàtica Industrial. Universitat Politècnica de València, Valencia, Spain; 2 Fent Innovative Software Solutions S.L., Valencia, Spain; King Abdulaziz University, SAUDI ARABIA

## Abstract

This paper addresses the problem of energy management of mixed criticality applications in a multi-core partitioned architecture. Instead of focusing on new scheduling algorithms to adjust frequency in order to save energy, we propose a partition to CPU allocation that takes into account not only the different frequencies at which the CPU can operate but the level of criticality of the partitions. The goal is to provide a set of pre-calculated allocations, called profiles, so at run time the system can switch to different modes depending on the battery level. These profiles achieve different levels of energy saving and performance applying different strategies. We also present a comparison in terms of energy saving of the most used bin-packing algorithms for partition allocation. As this is an heuristic, it is not possible to ensure that our results involve the minimum energy consumption. For this reason, we also provide a comparison with a exact method, such as constraint programming.

## Introduction

In real-time systems, there is an increasingly important trend for using applications with different levels of criticality where multiple components with different dependability and real-time constraints are integrated into a shared computing platform [1]. The reasons behind the trend for mixed-criticality are mainly non-functional: it reduces costs, volume, weight and power consumption, and can be found in a multitude of different domains such as industrial control, airborne, automotive systems and space avionics, only to cite the most notable ones.

At the same time mixed-criticality systems (MCS) are proliferating, computing platforms are migrating from single cores to multi-core architectures [2, 3]. It is estimated that multi-cores will be used in about 50% industrial applications by 2017. Multi-cores open new opportunities to develop robust mixed-criticality systems at competitive price but, on the other hand, scheduling in multi-core systems is significantly more complex than in a mono-core system. The theory that exists about this field, demonstrates that the problem of scheduling real-time tasks on a multi-core processor is NP-Hard [4], but apart from increasing the complexity, it also brings up new possibilities for the embedded applications.

Benefits of real-time scheduling and schedulability analysis for multi-core systems involve providing guarantees for temporal feasibility of tasks execution in advance. Schedulability analysis checks the time constraints of system tasks, provides a proper schedule, and significantly increases the efficiency of design and implementation of real-time systems [5].

In many domains such as avionics, space or industrial control systems, hard real-time constraints, safety and security issues and certification assurance levels are commonly required. Integrated Modular Avionics (IMA) [6] was an architectural proposal emerged as a design concept to integrate in a hardware platform several applications with different levels of criticality. IMA approach proposes to encapsulate functions into partitions configuring a partitioned system. Partitioned architectures isolate software components into independent partitions whose execution shall not interfere with other partitions, preserving temporal and spatial isolation. A spatial isolation protects the memory of a partition. Partitions are allocated in independent physical memory addresses. Thus, a partition can only access its memory areas. Temporal isolation means that only one application at a time has access to the system resources, making it impossible for an application to run when another application is running. In order to simplify the problem of shared resources among cores, we assume that the interference between cores is treated, in terms of time, as a part of the worst case execution time (wcet) of each task.

From a software architecture point of view, there is a trend in using virtualization techniques to provide a partitioning architecture under the temporal and spatial isolation. This approach was initiated in the avionics sector [7] and extended to space [8] and automotive [9]. Virtualization support for partitioning is provided by hypervisors. Hypervisors are layers of software that exploit the features of the hardware platform to establish independent execution environments, so that mixed-critical applications on a multi-core platform can be certified separately. Using hypervisors does not only include independence in terms of time and space, but also in terms of power consumption. Available energy for a multi-core system has to be shared by all running applications (i.e. increased power consumption of one application may reduce the available energy for other applications).

Energy management is also a very active research area in the recent years. Many approaches have been proposed to minimize energy consumption under the constraint that all tasks meet their deadlines. Most of the efforts have focused on new on-line scheduling algorithms which find an appropriate trade-off between the total utilization of the CPU and the energy consumption while meeting all deadlines. Two widely used techniques for reducing energy consumption are Dynamic Voltage and Frequency Scaling (DVFS) and Dynamic Power Management (DPM) [10]. For partitioned systems, these techniques can be used in their offline versions and reclaiming the static slack. The reason is that in a partitioned system, the scheduling plan is computed offline as defined in ARINC-653 [11]. Moreover, task migrations are not allowed because of the hardware state pollution caused and the consequent additional execution time overheads introduced [12, 13].

To generate a schedule plan in a multi-core partitioned system, the following steps are defined:

1. Allocation of workload to cores.
2. For each core, perform the schedule generation.

Energy management techniques have focused on Step 2, reclaiming the unused slack for scaling speed to save energy. Very few articles have addressed energy management in MCS, but almost none have studied the influence of the allocation algorithm in the energy consumption of the resulting schedule. That is why we are going to focus on Step 1. It is key to further consider the impact of task mapping on energy minimization.

Exacts methods, as linear programming solutions, are commonly used in order to solve a minimization problem. This implies that the approach presented in this paper does not offer the maximum energy savings by comparison with exact methods but we get faster execution time. In this paper, we also provide a comparison between our heuristic and a constraint programming solution, in terms of energy savings and execution time of both solvers.

### Contributions

This paper addresses the problem of energy management of mixed criticality applications in a multi-core partitioned architecture from the point of view of the allocation of workload to cores. We propose an allocation technique based on well-known bin packing algorithms that takes into account the different frequencies at which a core can operate.

Firstly, we will develop the technique for generic real-time systems (no MCS) and then we will add the consideration of MCS.

Some multicore systems can support an independent local clock per core. In this situation, *n* partitions can be running at different frequencies in different *n* cores simultaneously. Likewise, within a core, two or more partitions can run at different frequencies. But, once the frequency of a partition has been selected, it will remain constant during all system execution. The reason is that our study selects frequencies per partition in a static way, to give to the system (hypervisor or a supervisor partition) the information of partitions and frequencies. Changing frequencies in a core produces overheads to take account into the study. To simplify the problem, we do not consider this added cost in this paper but it is clear that we will treat it in the future work. Moreover, shared resources and communication channels between partitions must be also considered but, again for clarity purposes, have been avoided.

The goal of this paper is to provide a set of plans with different consumptions and performances, depending on how low-level criticality load is treated. Then, at runtime, the system will decide which plan to execute depending on, for example, the battery level.

The comparison with a exact method is also presented in this paper. We easily deduce that our method is not as exact in terms of energy savings but the time used to find the solution is even more drastically reduced.

### Related work

As part of the recent energy management research, several papers proposed DVFS-based solutions for real-time embedded systems running on conventional multi-processor platforms.

Recently, [14] presented a survey of energy management techniques for embedded systems, while Bambagini et al. [10] presented a survey focused on hard real-time systems.

Regarding energy management in multiprocessors, Xu et al. [15] proposed a heuristic to find the allocation of parallel tasks that minimizes energy the consumption. This work is similar to our proposal in the sense that is focused in the allocation of tasks to processors rather than in the online scheduling. However, their work applies to parallel tasks running on identical processors and it do not consider MCS. Devadas and Aydin [16] presented a system-level energy management of a set of processing cores that share the same supply voltage and frequency (voltage island).

Energy management in mixed-criticality multiprocessor real-time systems has been introduced in very few papers. The first paper that considers energy as important as schedulability in MCS is [17]. In this paper, the authors claim that energy is as important as time in mixed-criticality systems and they demonstrate how an incorrect handling of energy can violate mixed-criticality guarantees. They also propose an energy-aware mixed-criticality scheduler. In [18] a monitoring and control mechanism based on event-driven power estimation to isolate dynamic power consumption of mixed-critical applications running on a many-core platform is presented. In [19] the authors propose a scheduling algorithm that handles tasks with high-criticality levels such that no deadline is missed. For tasks with low-criticality levels, it finds an appropriate trade-off between the number of missed deadlines and their energy consumption. In [20] a DVFS technique is proposed for MCS together with an energy-aware task allocation on identical multi-core processor.

One work that focuses on energy efficient allocation in MCS is presented by Awan et al. in [21]. They present an approach to generate a set of feasible allocations and select the one with the lowest energy consumption. They assume the Vestal model for MCS. They also do not take into account the different frequencies of the cores in the allocation algorithm.

In [20], authors propose an energy saving method for MCS on multicore based on the isolation of tasks depending on their criticality levels on different cores. In our study, we have consider the mix of workloads from different criticality levels in different cores.

Seeing the publication dates of the articles, it is clear that it is a topic of interest where there are many open issues to solve.

## Methods

### Model

#### Task model

We consider a set of *m* heterogeneous cores *M*_1_‥*M*_*m*_. Each core can execute with any *g* frequencies independently, within the range [*f*_1_, *f*_*g*_], being *f*_*g*_ the highest frequency of each core. As cores are heterogeneous, each of them works in a different range of frequencies. However, for simplicity and to be able to adapt our model to the Safepower project [22], we suppose that all cores work in the same range of frequencies. As bin-packing algorithms are not influenced by system frequencies, this simplification does not affect to rest of the paper.

Each core is assigned a set of *p* critical partitions *P*_1_‥*P*_*p*_ that are defined by the pair *P*_*i*_ = (*τ*, L), where *τ* is the set of tasks and L is the criticality level of the partition.

We define two criticality levels per partition [22]: HI (high) and LO (low). All tasks that belong to the same partition have the same criticality level. HI partitions have to be executed to completion in any condition and temporal constraints of their tasks have to be fulfilled. LO partitions can be executed in some conditions. No temporal constraints are identified but it is expected a bandwidth for them. If, in some cases, they can be dropped, we call them disposable LO partitions (DLO). If they have to be executed in any case they are called required LO partitions (RLO). But, even if these partitions cannot be dropped, their bandwidth can be reduced if needed (for energy saving purposes). Dropping and other operations will be introduced later.

Each partition *P*_*i*_ is composed of *n*_*i*_ tasks $\tau_{i}=\left(\tau_{i1}‥\tau_{in_{i}}\right)$. Each task *τ*_*ij*_ is characterized depending on its criticality:

- HI tasks are defined as *τ*_*ij*_ = (*C*_*ij*_, *D*_*ij*_, *T*_*ij*_) where *T*_*ij*_ is the period, *D*_*ij*_ is the deadline and it is supposed to be equal to *T*_*ij*_ and *C*_*ij*_ is the active worst case execution time. Active worst case execution time of a task depends on the running processor frequency. Thus, computation time of each task is denoted as an array $\left[C_{ij}^{f_{1}},‥,C_{ij}^{f_{g}}\right]$ in which $C_{ij}^{f_{i}}$ is the worst case execution time (wcet) estimated at frequency *f*_*i*_. There is no need that all tasks spend their worst case time in all executions. Thus, we consider the most critical value (wcet) to do the analysis and it will ensure that any other value will be supported by our study.
  The utilization of a task *τ*_*ij*_ running at frequency *f*_*i*_ is $U_{ij}^{f_{i}}=\frac{C_{ij}^{f_{i}}}{T_{ij}}$.
- LO tasks *τ*_*ij*_ are characterized by their bandwidth $\eta_{ij}=\frac{B_{ij}^{f_{i}}}{T_{ij}}$, where $B_{ij}^{f_{i}}$ is the budget or worst case execution time at frequency *f*_*i*_ and *T*_*ij*_ is the period of a task *τ*_*ij*_. The utilization $U_{ij}^{f_{i}}$ of a task *τ*_*ij*_ running at frequency *f*_*i*_ is *η*_*ij*_.

We may assume without loss of generality that all preemptions occur at integer time values. We then assume, for the remainder of the article, that all parameters are indeed integers. Denote that the internal partition context switch in taken into account in the task computation. Moreover, core migration of a partition or a task is not allowed.

Utilization of a partition running at frequency *f*_*i*_ is given by $U_{i}^{f_{i}}=\sum_{j=1}^{n_{i}}U_{ij}^{f_{i}}$ being $U_{ij}^{f_{i}}$ defined previously.

Regarding to the distribution of tasks and partitions to cores, methods presented in this paper solve both situations:

- All tasks belonging to the same partition are assigned to the same core. In this situation, the unit which is allocated to cores is the partition.
- Tasks of the same partition are assigned to different cores. In this situation, the unit which is allocated to cores is the task.

Henceforth we will assume that all tasks in a partition are assigned to the same core. The reason is to avoid overheads. Thus, partitions will be allocated to cores and the total core utilization $U_{C_{i}}$ is defined as the sum of all utilizations of partitions assigned to that core *C*_*i*_.

Note that, from the criticality point of view, our model is simpler than Vestal’s model [23] in the sense that we assume the longest execution time observed in testing as a unique WCET estimate for each frequency. The reason is to avoid having a computation times matrix (two dimensions for frequency and criticality level). Vestal’s model also use the same term “criticality” to refer both to the criticality of a task and the mode of operation [24]. We consider that the system has different operational modes each one associated a static schedule. In Vestal’s model, two different operational modes will differ only in the computation times. In our model (based on ARINC-653 Part 2), each operational mode can have associated a different set of tasks.

#### Power model

Power consumption is divided in dynamic consumption and static consumption. The former depends on the activity of the processor while the latter is mainly due to leakage current and can only be reduced by activating a low-power state. We will assume the power model from [20]:

$$\begin{array}{c} P\left(f\right)=P_{s}+\beta f^{\alpha } \end{array}$$

where *P*(*f*) is the total power consumption at operating frequency *f*, *P*_*s*_ is the static power consumption and *βf*^*α*^ is the dynamic power consumption. A common assumption is that 2 ≤ *α* ≤ 3.

### Energy efficient partition allocation in non MCS

In next sections, we are going to present our solution to the mixed-criticality energy minimization problem (from the allocation point of view). This solution is based on the utilization of an allocation heuristic. Allocation to cores can be solved using a bin-packing algorithm. The approach requires to define objects (partition utilizations *U*_*i*_), which can then be packed on to the bins (cores).

Among all these heuristics, Worst Fit Decreasing Utilization (WFDU) is known to obtain a well-balanced load among cores. In [25] it is demonstrated that this balance-load also minimizes energy consumption. But this statement is done for systems with applications running at the same frequency.

#### Motivational example

This example is taken from [25] adapted for our partition model. Consider four partitions with *U*_1_ = 0.5, *U*_2_ = 0.4, *U*_3_ = 0.4 and *U*_4_ = 0.3 running on *m* = 2 heterogeneous processors. By [25] we know that the allocation that minimizes energy is $U_{C_{1}}=U_{1}+U_{4}=0.8$ and $U_{C_{2}}=U_{2}+U_{3}=0.8$ since it is the most balanced one. But this is considering that all partitions run at the same frequency. Following the model defined before, with *g* = 2 frequencies (*f*1, *f*2) then each partitions will have 2 possible utilizations. The values are shown in Table 1. Now, allocators can choose one of the two utilizations for each partition. But which one of all possible combinations results in the most energy efficient?.

**Table 1:** Utilizations.

	$U_{i}^{f1}$	$U_{i}^{f2}$
*P*_1_	0.7	0.5
*P*_2_	0.56	0.4
*P*_3_	0.56	0.4
*P*_4_	0.42	0.3

In this section an energy efficient partition allocation is presented in which each partition can run at a different frequency. This frequency will not change throughout the system execution. Making an analogy with priorities, DVFS will treat frequencies as dynamic priorities while our method considers frequencies as static priorities. However, our method does not prevent any further dynamic slack reclaiming. Our proposal is not optimal, since computing the allocation that minimizes overall energy consumption is intractable.

Algorithm 1 shows the EEA (Energy Efficient Allocator) algorithm to allocate partitions to cores.

**Algorithm 1**: Allocation for energy management

**Data**: Allocator, sorting

**Result**: mapping

1 **Function** *EEA (Allocator, sorting)*

2  k → 0;

3  mapping ← core workload;

4  *Set all partition frequencies to the maximum frequency*;

5  **Loop**

6   mapping(k) = Allocate (Allocator);

7   **if** *feasible* **then**

8    k → k+1;

9    *P*_*i*_ = selectPartition(sorting);

10    **if** *P*_*i*_ = −1 **then**

11     exit;

12    **end**

13    decreaseFrequency(*P*_*i*_);

14   **else**

15    increaseFrequency(*P*_*i*_);

16    exit;

17   **end**

**Algorithm 2**: Partition selection

**Data**: sorting

**Result**: Allocated partition or not

1 **Function** *selectPartition (sorting)*

2  *sort partitions according to sorting criteria to create an array of heaps Ulist*;

3  **for** *i* = *g*; *i* > 2; *i* = *i* + 1 **do**

4   p = Head(UList(i));

5   **if** *p*! = *null* **then**

6    return p;

7   **end**

8  **end**

9  return -1;

In this algorithm we can choose the type of allocator and the criterion with which the partitions are selected to decrease its frequency. As allocators we have considered WFDU, FFDU and BFDU (the same ones that have been compared in [25]). As sorting criteria, partitions can be chosen by decreasing utilization (DU), increasing utilization (IU) or randomly (R). This way we can make a comparison between these allocations algorithms and know which of them is better for energy management purposes.

The algorithm starts assigning the highest frequency to all partitions. The allocation of this partition set (*k* = 0) is called the original mapping that is also the mapping with the most energy consumption. Then, a partition is selected (following the criteria DU, IU or R) to decrease its frequency to *f*_*g*−1_ and an allocation is again performed (*k* = 1). The algorithm runs sequentially decreasing partition frequencies until we reach a non-feasible mapping or all the partitions have reached the minimum frequency. We consider a feasible mapping if the utilization of each core is below 1 [26].

The frequency decrease works as follows (Algorithm 1): for each frequency *f* there is a heap of partitions that have assigned this frequency sorted according to the sorting criteria. The partition to decrease its frequency is chosen from the head of the list of the highest frequency. If the only non-empty heap is the one with frequency equal to *f*_1_ the function *selectPartition* returns -1 and the algorithm stops since all partitions have reached their lowest frequency.

The result of this algorithm is a mapping with the minimum consumption before the system becomes unfeasible. In addition, each iteration of the loop gets a mapping with less consumption than the previous one. So, we can compare bin-packing algorithms by energy consumption of the last mapping and parameter *k* that is the number of mappings obtained by the algorithm.

#### Example

**Fig 1:**
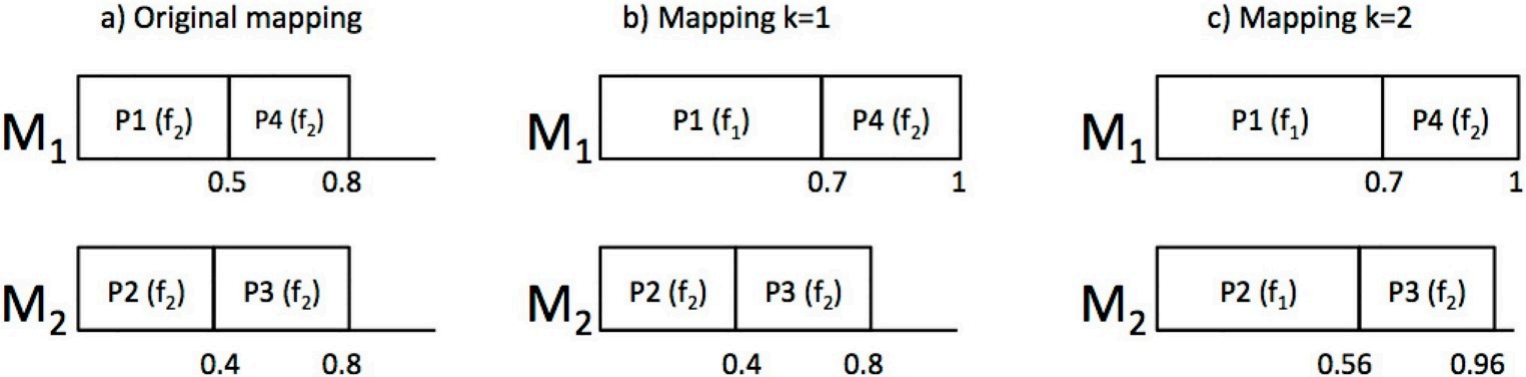
EEA mappings.

Back to the motivational example of this section, Fig 1 shows the iterations of the EEA algorithm. We have run WFDU2, that is, partitions have been allocated following WFDU and the partition selection criteria is by decreasing utilization. Fig 1A corresponds to the original mapping, in which all partitions run at the maximum frequency *f*2. In the first iteration, P1 is selected to decrease its frequency, so we change its utilization from $U_{1}^{f2}=0.5$ to $U_{1}^{f1}=0.7$. The result is shown in Fig 1B. In the next iteration (k = 2), P2 is selected to decrease its frequency resulting in Fig 1C. The algorithm stops because the next mapping will cause $U_{C_{2}}>1$.

For the calculation of the energy consumption, we assume *α* = 3, *β* = 1 and *P*_*s*_ = 0.8*W* [20]. The processor frequencies are [*f*_1_, *f*_2_] = [0.8, 1.1]*GHz*. The energy consumption for an hyperperiod *H* is calculated as *E* = *P*(*f*)*H*. The results are shown in Table 2. As we see, increasing the total utilization of the cores by decreasing the frequencies at which partitions run results in an energy saving with respect to the original mapping up to a 8% in this simple example.

**Table 2:** Energy consumptions of the mappings of the example.

	*M*_1_	*M*_2_	Total
Mapping a)	170.48	170.48	340.96
Mapping b)	155.77	170.48	326.25
Mapping c)	155.77	158.71	314.48

### Partition allocation in MCS

Once the energy efficient allocation method has been presented, in this section we are going to complete the proposal with the inclusion of mixed-criticality systems.

In the previous section all partitions have the same criticality level, but when partitions have different levels of criticality, we can follow the next strategies to save energy:

- Trim the bandwidth of RLO partitions. RLO partitions cannot be dropped but their bandwidth can be reduced. We propose the reduction in the following way:
  For a RLO partition *P*_*i*_ with $U_{i}^{f_{1}},‥,U_{i}^{f_{g}}$, it is trimmed when it executes at the lowest frequency *f*_1_ but with the utilization of the highest frequency *f*_*g*_. In practice, this is possible due to the utilization of a static cyclic scheduler in which we can force the duration of the slots. This way we can assign $C_{ij}^{f_{g}}$ units of time but running at frequency *f*_1_.
  Let’s assume that for a time window of length the hyperperiod *H*, the units of time that a partition *P*_*i*_ executes under frequency *f*_1_ is:
  $$\begin{array}{c} H\frac{C_{i}^{f_{1}}}{T_{i}}=HU_{i}^{f_{1}} \end{array}$$ (1)
  When a partition is trimmed, it executes
  $$\begin{array}{c} H\frac{C_{i}^{f_{g}}}{T_{i}}=HU_{i}^{f_{g}} \end{array}$$ (2)
  Therefore, there is a performance loss of:
  $$\begin{array}{c} 1-\frac{U_{i}^{f_{g}}}{U_{i}^{f_{1}}}=1-\sum_{j=1}^{j=n_{i}}\frac{C_{ij}^{f_{g}}}{C_{ij}^{f_{1}}} \end{array}$$ (3)
- Trim the bandwidth of DLO partitions. This is done in the same way as for RLO partitions. The performance loss will follow the same equation.
- Drop DLO partitions. As stated in previous sections, these partitions can be dropped if needed. In this case, the performance loss of this partition is 1.

To summarize, dropping a partition means not running it, while trimming is an intermediate solution between executing completely the partition and not executing it at all. A trimmed partition executes at its higher frequency but with the lowest WCET. It is indicated for partitions whose activities are “best effort”, that is, partitions with soft timing constrains, as they are a LOW criticality partition.

Depending on how we combine these strategies, we will obtain different mappings with different grades of energy consumption and performance. The idea is to trim and/or to drop RLO/DLO partitions and execute EEA algorithm, that will use the free utilization of DLO and/or RLO partitions to decrease frequencies as much as possible. Then, we are going to define the profiles, taking into account that each profile is a consequence of the previous profile, i.e., adds changes to the previous actions. We propose the following profiles:

- Profile 1: Maximum energy saving without performance loss. In this case, we do not allow to drop any DLO partition neither trimming RLO partitions. Therefore, this is equivalent to treat all RLO and HI partitions as HI partitions and the EEA algorithm is executed as explained before.
- Profile 2: This profile will only allow trimming DLO partitions.
- Profile 3: This profile will allow trimming RLO partitions.
- Profile 4: In this profile we drop all DLO partitions.
- Profile 5: In this profile we execute the EEA algorithm in HI partitions as much as possible.

It is clear that as long as the number of the profile increases, the energy saving and the performance loss also increases.

This profiles are different mappings in which each partition will run at a specific frequency. Then, each profile will derive into a static scheduling plan stored in the configuration file of the hypervisor. This way, each operational mode instead of having one scheduling plan associated, now it will have 5 scheduling plans. At runtime, a supervisor partition will be in charge of change the profile depending on the energy state of the system. This change always occurs when the major activation frame (MAF) is completed. This ensures that all tasks complete their execution before changing the operational mode. An example of the advantage of changing plans is to link them to the battery level. Profile 1 will operate when battery level is between 100-81%, Profile 2 with 80%-61%, Profile 3 with 60%-41%, Profile 4 with 40%-21% and Profile 5 with 20%-1%.

#### Example

Using the same example as before, now we will assume that *P*_1_ and *P*_2_ are HI partitions, while *P*_3_ is RLO and *P*_4_ is a DLO partition. Profile 1 is equivalent to the mapping obtained in Fig 1C.

From this starting point, we can derive the rest of the profiles:

- Profile 2: In this profile, the utilization of *P*_4_ for *f*_1_ is trimmed from 0.42 to 0.3.
- Profile 3: In this profile, the utilization of *P*_4_ for *f*_1_ is trimmed from 0.42 to 0.3 and the utilization of *P*_3_ for *f*_1_ is trimmed from 0.56 to 0.4.
- Profile 4: This profile does not contain *P*_4_.
- Profile 5: In this profile, *P*_4_ does not exist and the utilization of *P*_3_ for *f*_1_ is trimmed from 0.56 to 0.4.

**Fig 2:**
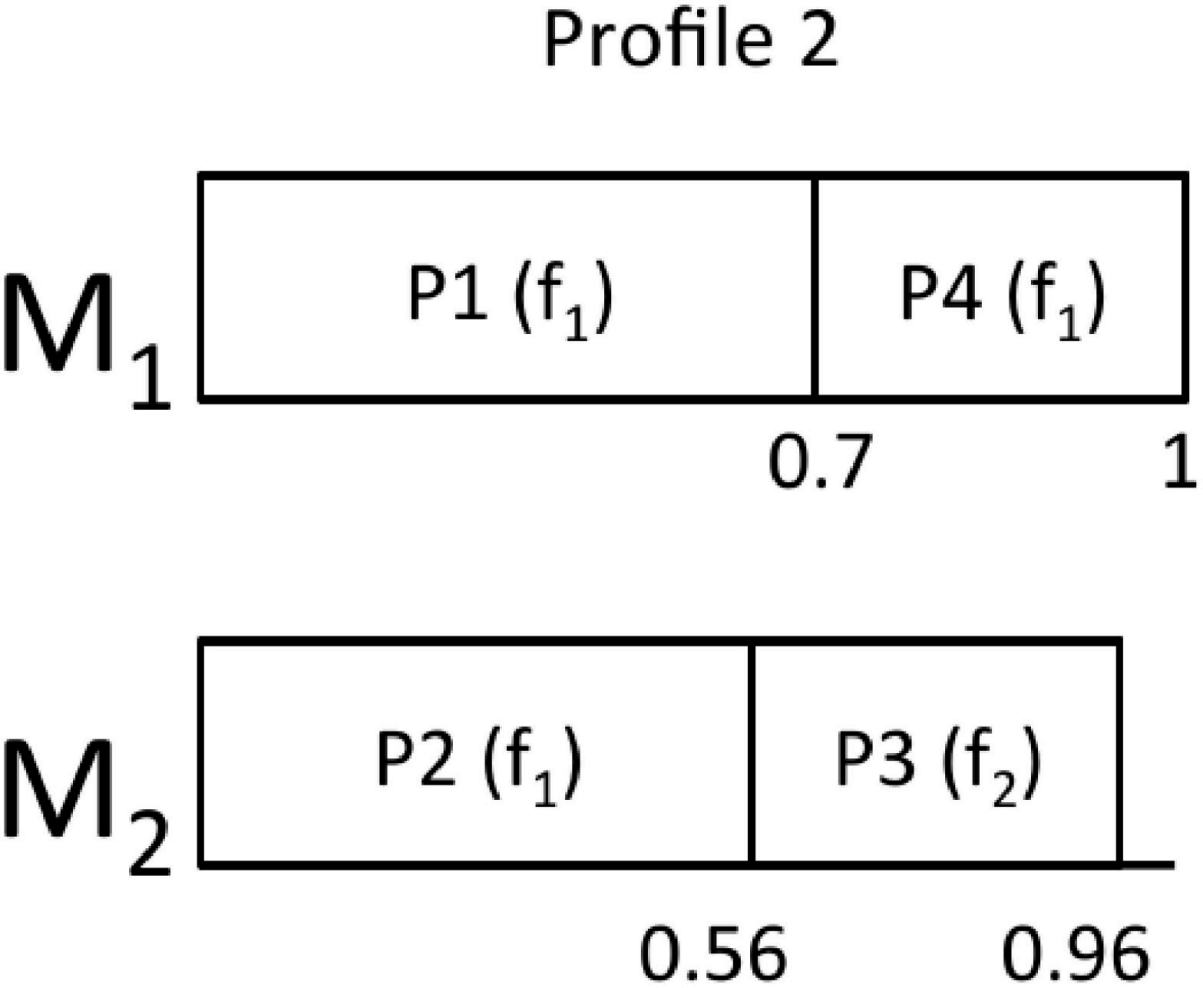
Mapping for Profile 2.

**Fig 3:**
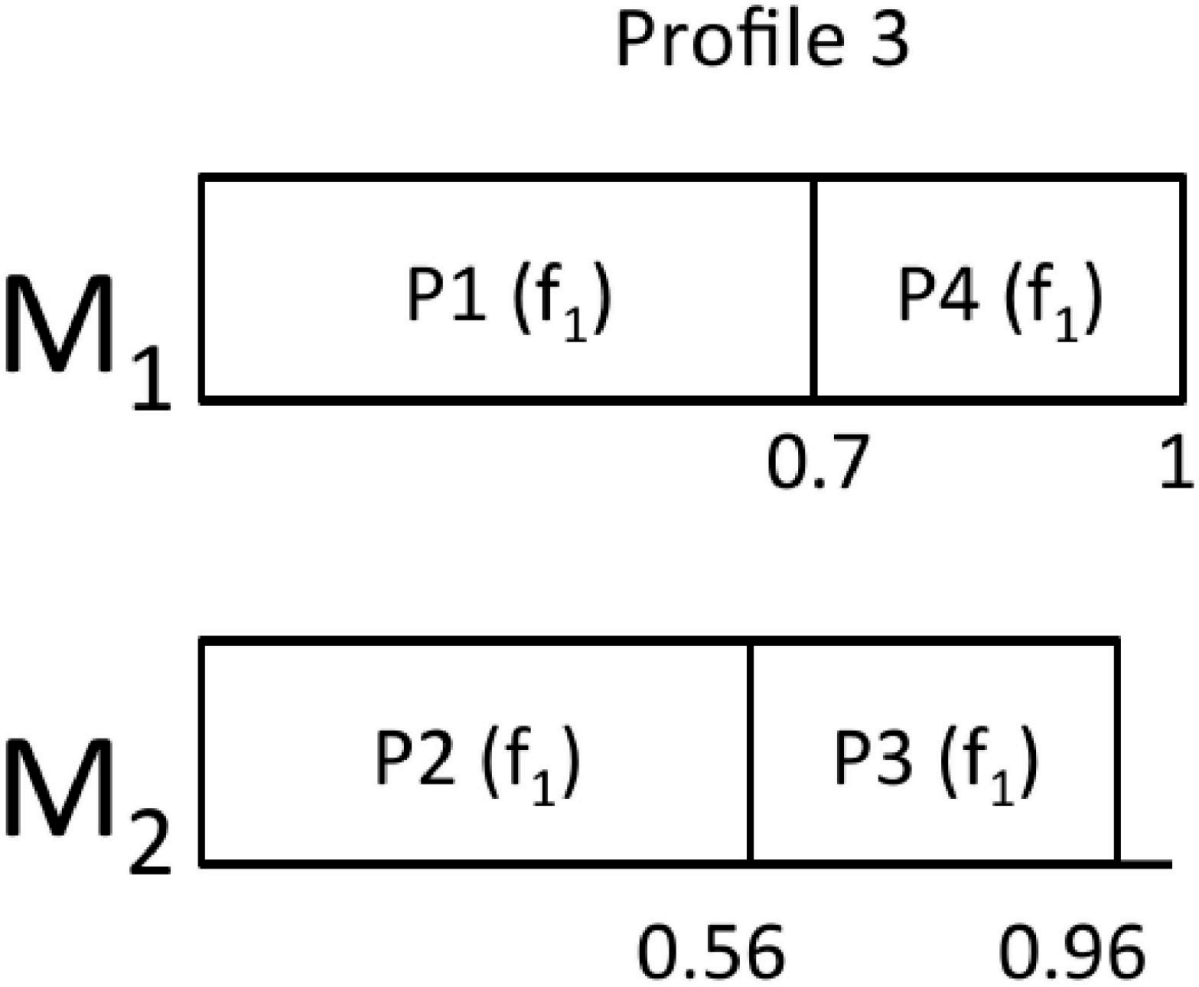
Mapping for Profile 3.

**Fig 4:**
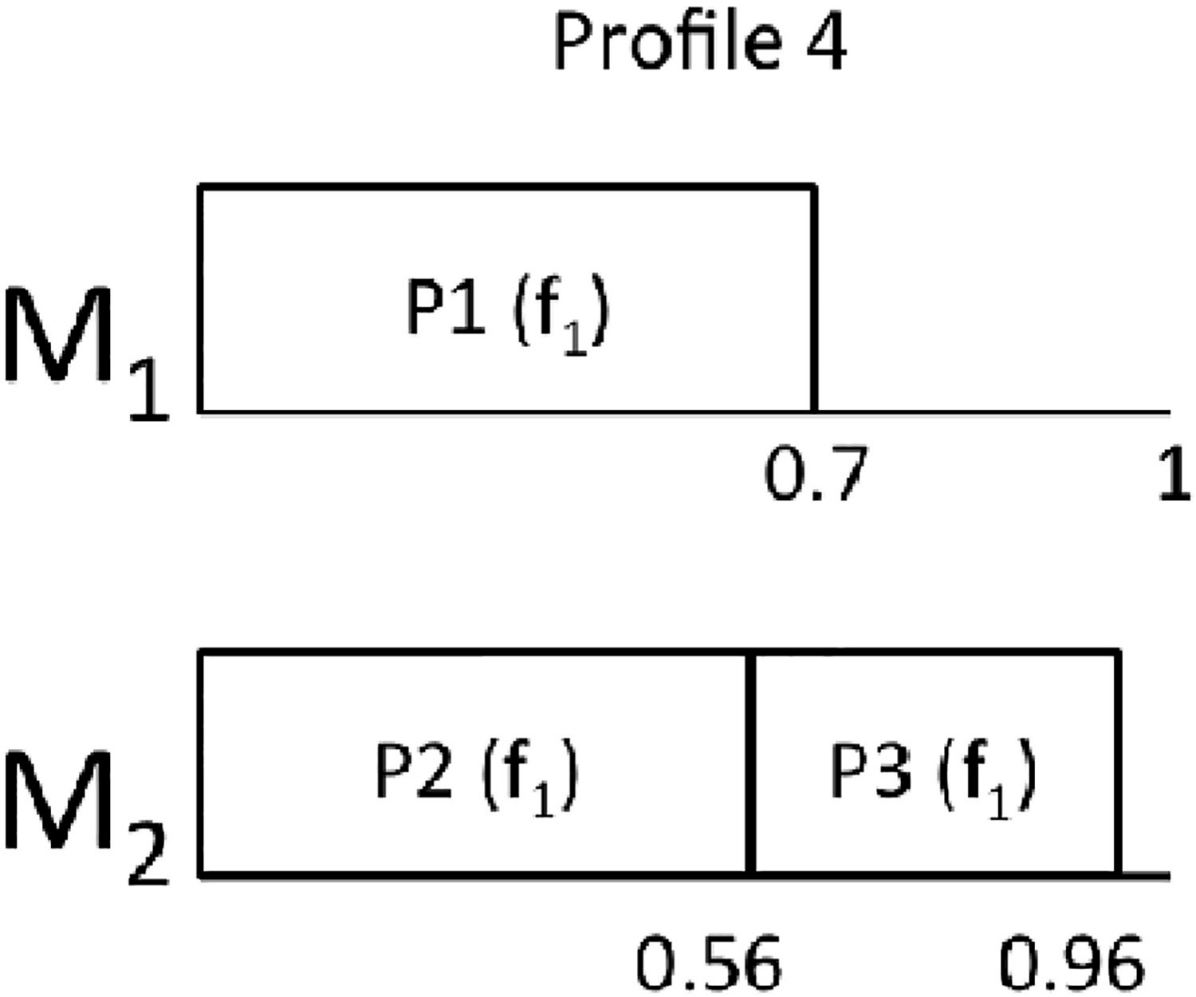
Mapping for Profile 4.

**Fig 5:**
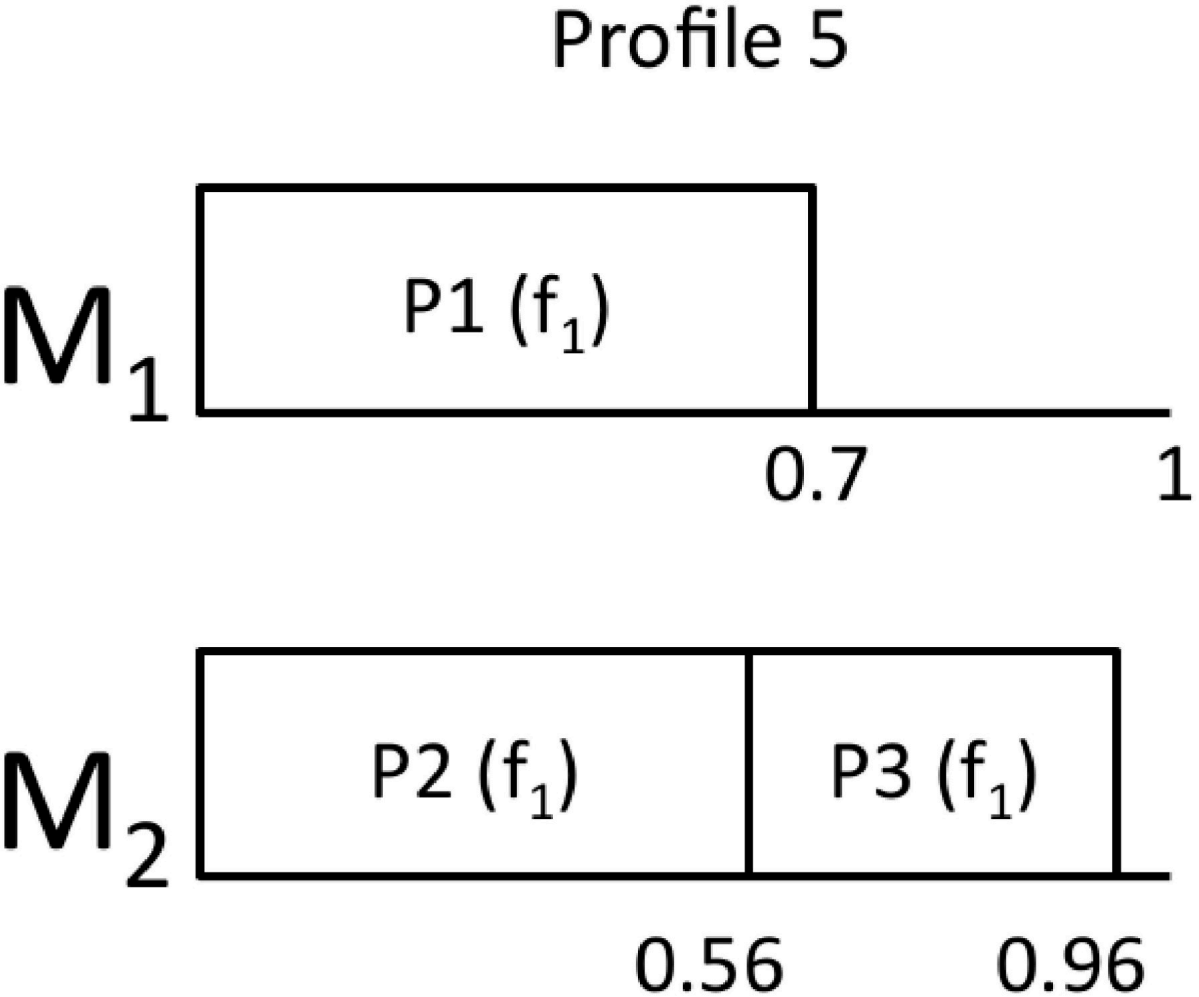
Mapping for Profile 5.

Figs 2, 3, 4 and 5 show the resulting mappings of the previous profiles. In Profiles 4 and 5, dropping partition *P*_5_ does not achieve any benefit because the extra “space” cannot be used to allocate any more partition in *M*_1_. The different energy consumptions are presented in Table 3. We can see how this time, the energy saving is more important at the expense of losing performance.

**Table 3:** Energy consumptions of the different profiles.

	*M*_1_	*M*_2_	Total
Profile 2	131.2	158.71	289.91
Profile 3	131.2	104.96	236.16
Profile 4	91.84	158.71	250.55
Profile 5	91.84	104.96	196.8

This performance loss is 0.25 for *P*_4_ trimming, 1 for *P*_4_ dropping. Regarding *P*_3_, performance loss is 0.28.

## Results

We developed a simulator to implement the proposed algorithms and a synthetic task and partition generator.

A number of tests have been run, specifically 10^5^ synthetic partition sets have been generated for *m* = 4 and total utilizations varying from 2.5 to 4 in steps of 0.1, resulting in 1500000 total simulations.

Each core utilization depends on the criticality level of the core, being the most critical cores those with highest utilization. The sum of all utilizations of cores is equal to the utilization of the system, defined by the user, with a maximum value of *m*.

The number of partitions for each criticality level is calculated as follows:

- HI level: a random value within the interval [4, 8].
- LO level (RLO): a random value within the interval [3, 6].
- LO level (DLO): a random value within the interval [3, 8].

Partitions utilizations were generated using the UUniFast algorithm [27] that gets an unbiased distribution of utilizations.

Regarding to tasks, the number of tasks per partition is calculated as follows:

- HI partitions: a random value within [2, 8] tasks per partition.
- LO level partitions (RLO): one task per partition.
- LO level partitions (DLO): one task per partition.

Tasks utilizations were also generated using the UUniFast algorithm [27]. Tasks parameters are calculated for the greatest frequency, *f*_*g*_. Once task utilization has been deduced, period is selected *randomly* in such a way that the hyperperiod of tasks is not a very big value. Then, the computation time of *τ*_*ij*_ at a frequency *f*_*g*_ is calculated as $C_{ij}^{f_{g}}=U_{ij}^{f_{g}}\cdot T_{ij}$.

Computation time $C_{ij}^{f_{i}}$ of a task *τ*_*ij*_ increases by reducing its frequency *f*_*i*_, i.e. the higher the frequency, the shorter the computing time. To simplify the problem, we suppose that this relation is linear. In particular, in the first step, computation times of tasks are generated with the greatest frequency. Consequently, to complete the array of values of wcet, we use the values of available system frequencies as follows:

**Definition 1** *Let us denote as f*_*p*_ *the value from which frequencies are greater or equal to 1 within the range* [*f*_1_, …, *f*_*p*_, …, *f*_*g*_]. *Then*:

$$\begin{array}{c} C_{ij}^{f_{m-1}}=\{\begin{array}{cc} \frac{C_{ij}^{f_{m}}}{f_{m}} & if\;0\leq m\leq p\\ C_{ij}^{f_{m}}\cdot f_{m} & if\;p+1\leq m\leq g \end{array} \end{array}$$ (4)

All these parameters are saved in an array and used for frequency changes in tasks. It is clear that linear relation is a simplification of the problem. If this relation is not known, the user will provide the array of computation times to make the work succeeds.

When everything is ready, we start the simulator to calculate different mappings with different grades of energy and performance, as was explained before.

As we mentioned in initial sections, there are some differences between choosing as allocation unit a task or a partition.

We have conducted the same experiments for 8 cores. In this case, the number of partitions have been multiplied by 2. Other experiments consist on using 2 cores. In both cases, similar results have been obtained as for 4 cores.

### Comparison of allocation methods in non MCS

In this section a comparison between the different allocators and partition selection is done. We measure two parameters:

- Energy saving: This is the saving of the final mapping with respect to the original mapping, that is, the mapping at which all partitions run at the highest frequency (iteration *k* = 0).
- Number of feasible mappings. This corresponds to the iterator *k* of Algorithm 1.

**Fig 6:**
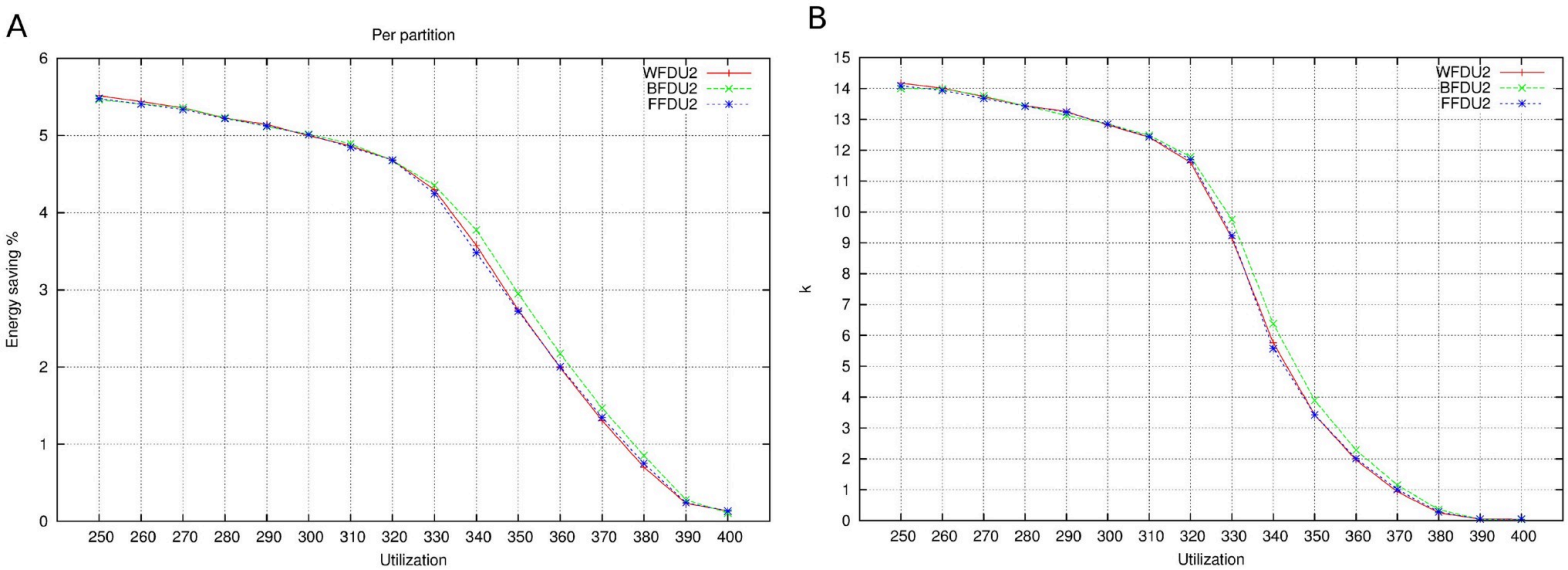
DU. A: Energy saving. B: Number of mappings.

**Fig 7:**
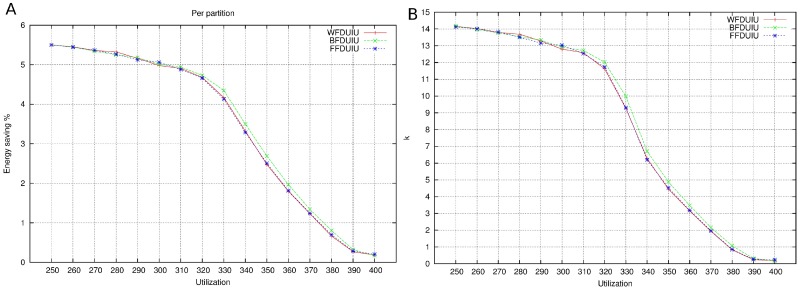
IU. A: Energy saving. B: Number of mappings.

**Fig 8:**
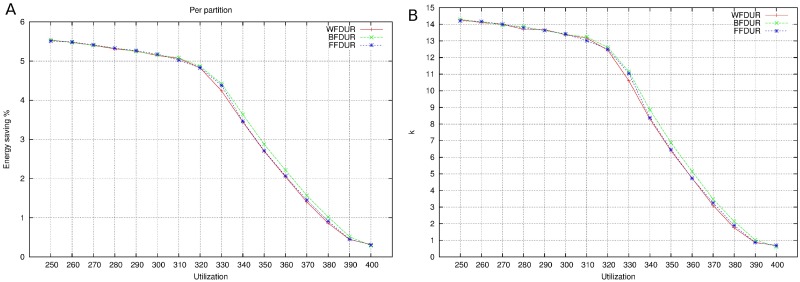
R. A: Energy saving. B: Number of mappings.

Figs 6A, 7A and 8A show the results for 4 cores. In this pictures, the relation between energy saving and utilization factor (from 240% to 400% in the case of 4 cores) is depicted. As utilization factor increases, it is observed that energy saving is reduced. When cores are almost full (utilization 380-400%), reducing frequency (i.e. increasing computation times) will make the system infeasible. For this reason, the scope of energy saving is short.

As it is seen in the figures, the three base bin packing algorithms present very similar results. This is not a surprise, since we are measuring the energy of the final mapping, which corresponds to a situation in which all the cores will be at full capacity. Moreover, there is also no difference in the partition selection criteria as far as energy saving is concerned.

However, the number of mappings is depicted in Figs 6B, 7B and 8B. It seems that WFDU is the algorithm that needs more iterations to reach the optimal solution and R is the worst partition selection criteria, due to the randomness of its results. It is clear that the more utilization factor, the less iterations are possible to perform.

**Fig 9:**
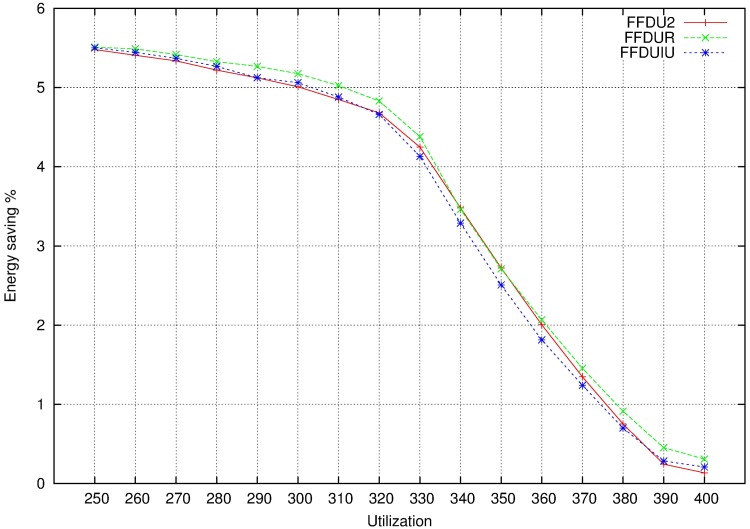
Energy saving no MCS when allocator is FFDU.

To avoid adding all the results in this paper, we show in Fig 9 the reason to select FFDU2 as allocator. Although we know that different allocators provide very similar results, FFDU2 allocator provides slightly better outcomes.

**Fig 10:**
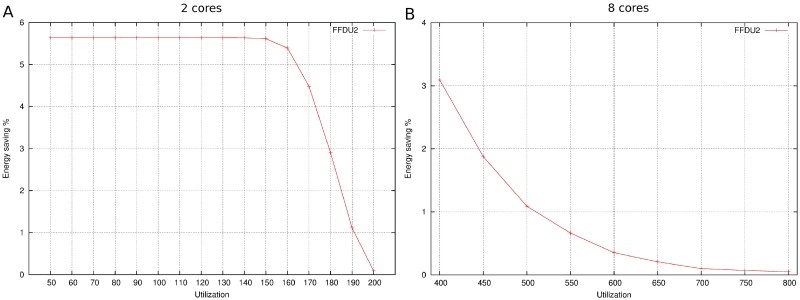
Energy saving with 2 and 8 cores. A: 2 cores. B: 8 cores.

Fig 10 depicts, as in previous figures, the relation between energy saving and utilization factor but, in this case, experiments have been developed in 2 (Fig 10A) and 8 cores (Fig 10B). It is demonstrated again that energy saving decreases with utilization factor, being almost zero when cores are getting full.

As different allocators provide similar results, let us complement the results section with a comparison between an exact method (constraint programming solutions, CP) and our heuristic. In almost all situations CP provides the same solution as our heuristic. But, in some scenarios, CP provides better energy savings than our algorithm. However, in terms of time consumptions, our algorithm offers much better results.

**Fig 11:**
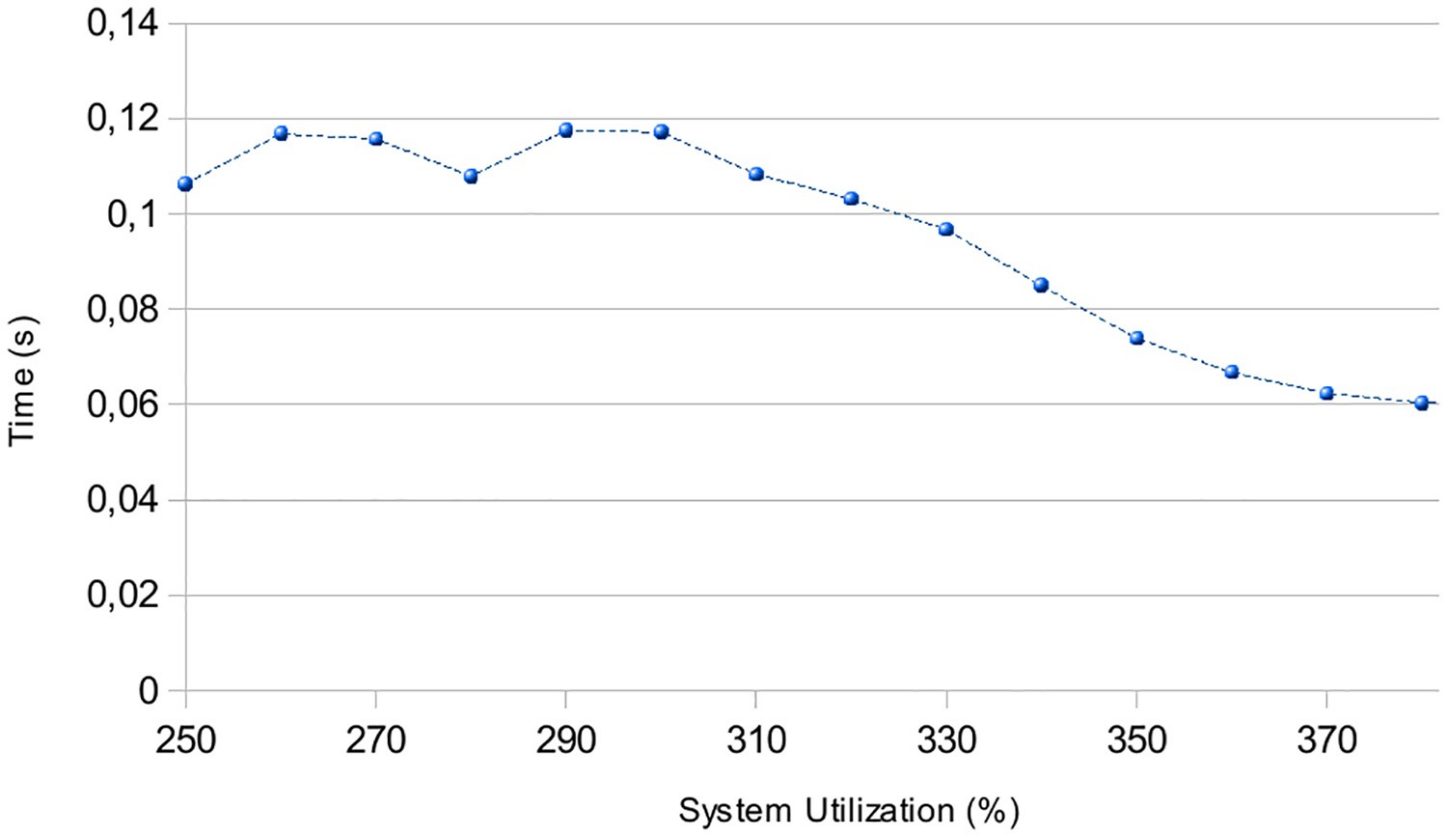
Time consumption in executing EEA algorithm.

Fig 11 depicts the average time used by the CPU in executing 50 iterations of the EEA algorithm. Each iteration consists of allocating a number between 10 and 20 partitions in 4 different cores, in order to minimize the energy consumption. The time measured in CP simulator is directly provided by the solver. The time in heuristic algorithm is calculated measuring the number of instructions and the frequency of the CPU. WE can obverse that the more the system utilization is, the less time the algorithm needs to reduce the system frequency (increasing system utilization is less possible).

We observe that only one experiment expends between 15 and 30 minutes and even more, depending on the system parameters, number of cores and partitions, number of available frequencies, etc. In a simple situation with 4 partitions allocated in 2 cores as in Table 4 with 2 system frequencies, the times used to solve the problem in each situation and energy savings are summarised in Table 5.

**Table 4:** Utilizations—Example.

	$U_{i}^{f1}$	$U_{i}^{f2}$
*P*_1_	0.62	0.5
*P*_2_	0.41	0.35
*P*_3_	0.69	0.58
*P*_4_	0.45	0.37

**Table 5:** Comparision between EEA and CP solvers.

	$E_{M_{1}}$ (Ws)	$E_{M_{2}}$ (Ws)	TOTAL ENERGY (Ws)	Simulation Time
EEA	10.4275	14.725	25.1525	0m0.202s
CP	12.6225	12.1125	24.735	14m 5s

In Table 5, it is observed that the energy consumption is bigger with EEA than CP, but the time the CP solver needs to find the solution is significantly higher.

In next subsection we use the same simulator in order to evaluate the situation in a mixed criticality system.

### Energy saving and performance loss in MCS

We conducted the same set of experiments to measure the energy saving achieved, the performance loss and number of mappings of the 5 profiles explained previously.

**Fig 12:**
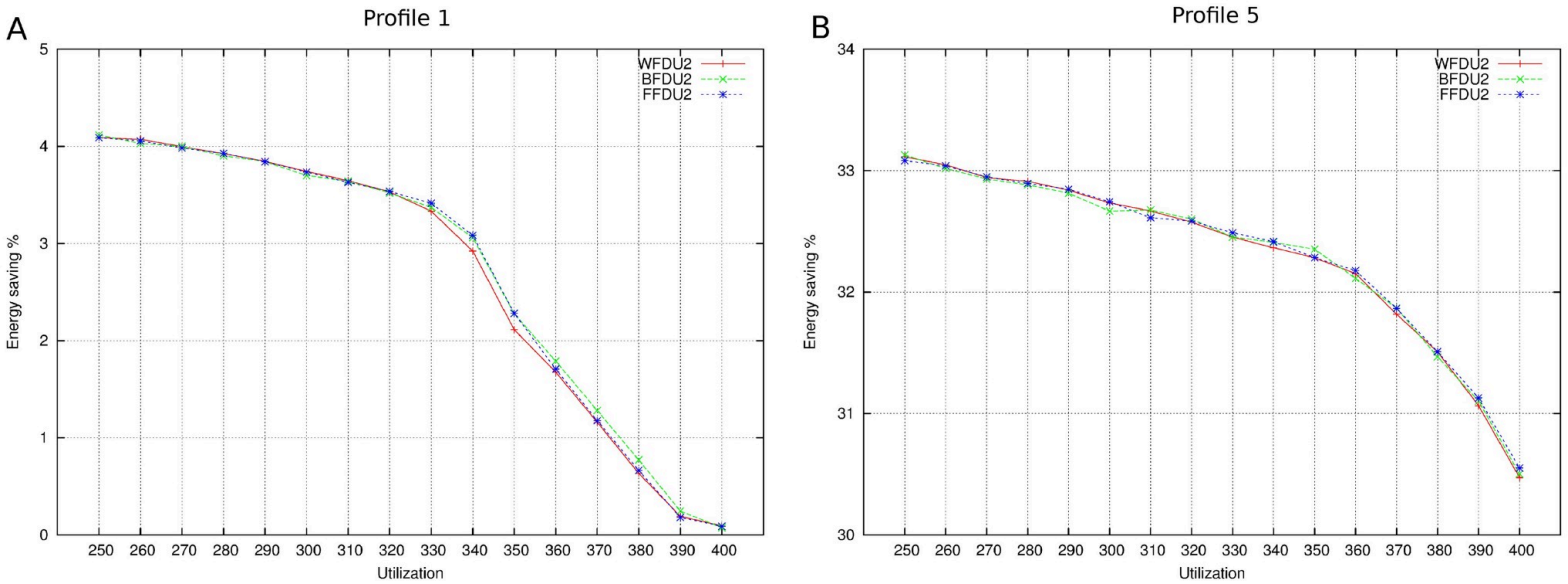
Energy saving with different allocators. A: Profile 1. B: Profile 5.

Fig 12 depicts energy savings with different allocators and profiles. It shows the more system utilization increases the more energy saving decreases. If cores are almost full, decreasing system frequency is becoming increasingly difficult and saving energy is also difficult.

**Fig 13:**
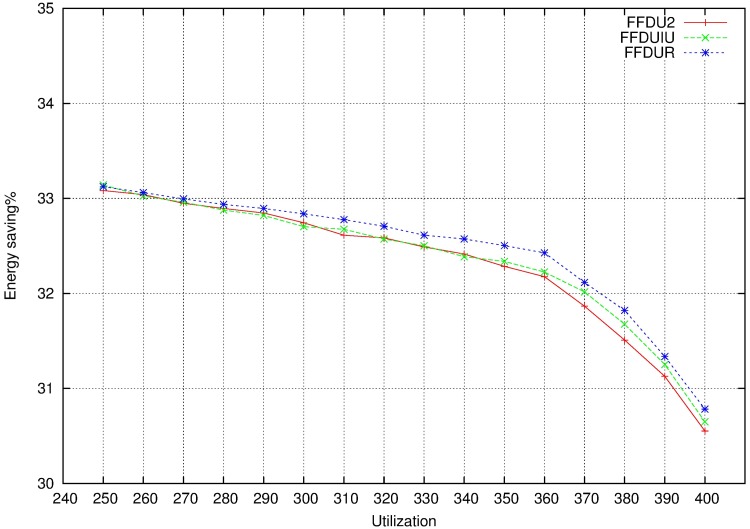
Energy saving MCS when allocator is FFDU.

As is no MCS, we show in Fig 13 the reason to select FFDU2 as allocator.

With FFDU2, we measure the parameters mentioned before: energy saving, performance loss and number of mappings.

**Fig 14:**
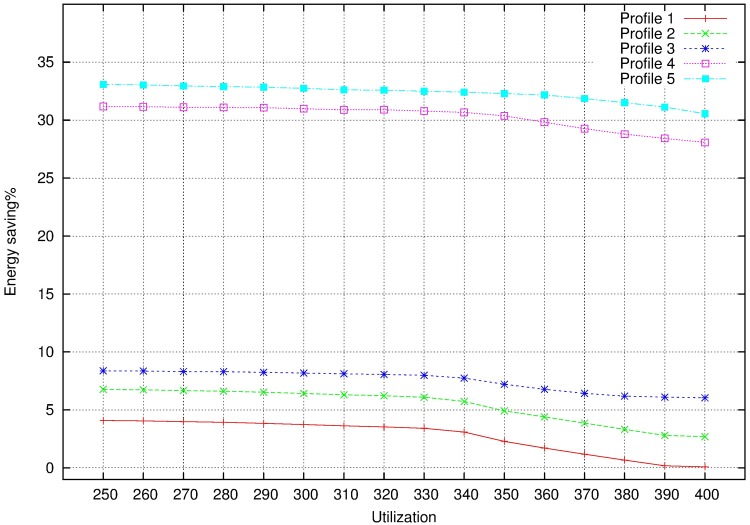
Energy saving for MCS profiles.

**Fig 15:**
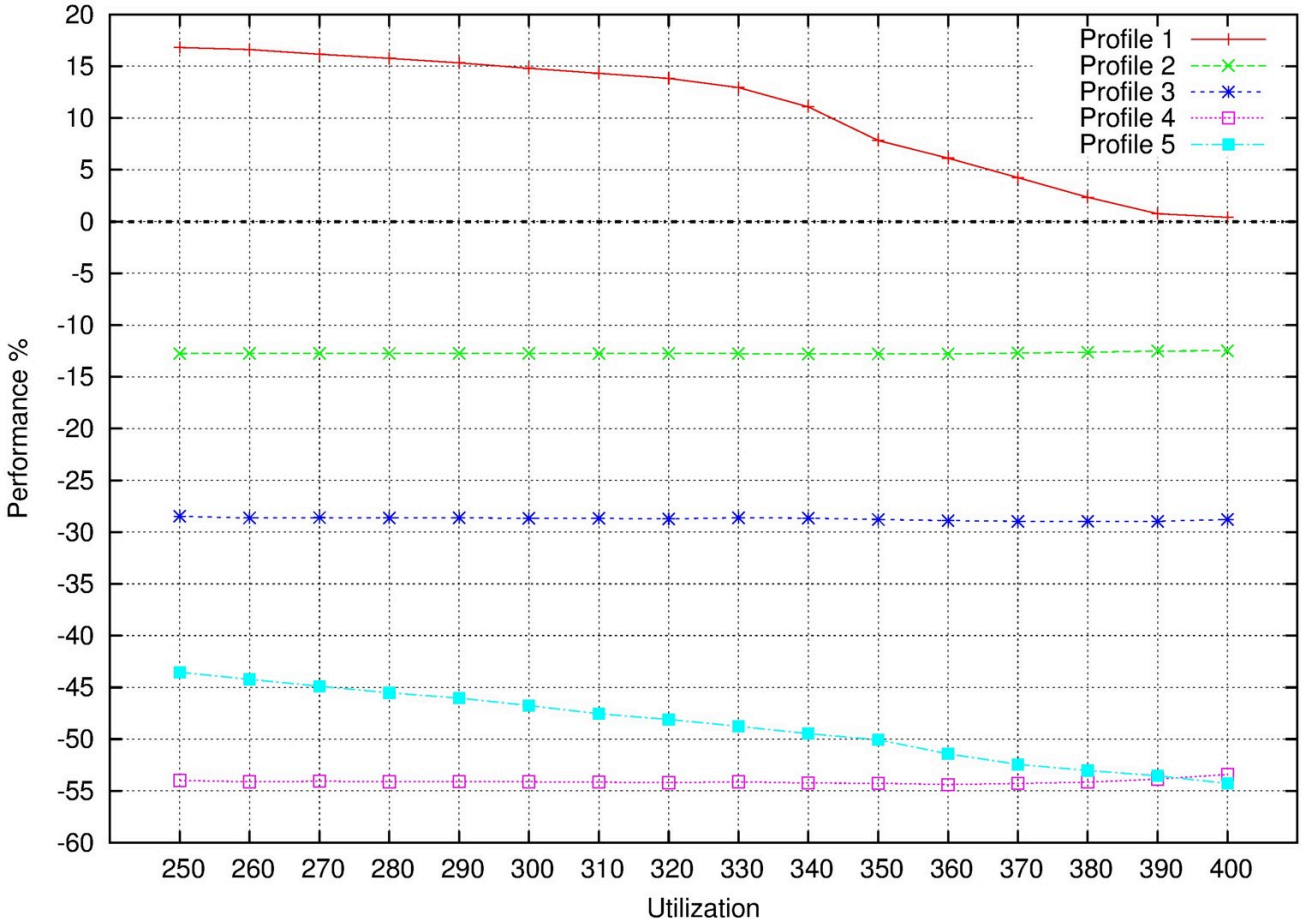
Performance loss for MCS profiles.

**Fig 16:**
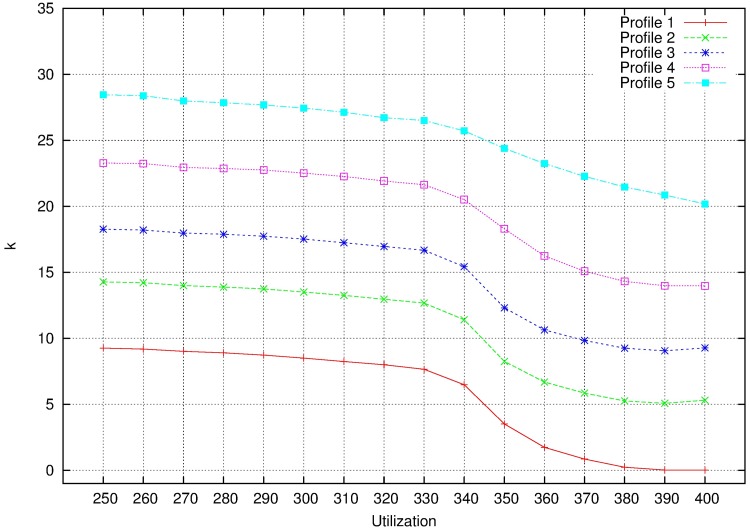
k for MCS profiles.

- Energy saving is measured as in non MCS. As each profile uses lower system frequency than the previous profile, energy saving will be greater as profile increases. It is depicted in Fig 14. When DLO tasks are dropped and the system frequency is reduced as much is possible (profile 5), energy saving is about 35%.
- Performance loss in profile *i* is calculated as the relation between the system execution times in profile *i* and profile 0, being profile 0 the original system at the maximum frequency. If we consider that profile 0 supposes 0% of performance loss (Fig 15):
  - Profile 1 increases the performance in relation to profile 0 (the system is 15% closer to be completely filled).
  - Profile 2 decreases the performance when DLO tasks are trimmed (performance loss of 10% with respect to profile 0)
  - Profile 3 also decreases the performance when RLO tasks are trimmed (performance loss of 30% with respect to profile 0)
  - Profile 3 also decreases the performance when DLO tasks are dropped (performance loss of 55% with respect to profile 0)
  - Profile 5 increases the performance in relation to profile 4 (increasing computation time of HI tasks by reducing the system frequency) but, with respect to the original profile, there is a performance loss of 40-55%.
- Number of mappings. Parameter k is calculated as in non MCS. As it is seen in Fig 16, obviously the number of mappings increases with profile. It is obvious that the more operations (k) to partitions are needed, the more attempts to fill the cores are done.

Using a CP solver in order to conduct these experiments provides worst solutions than in non MCS. This occurs because the number of constraints increases due to the addition of the criticality of the partition. In most cases, we have spent several days without reaching the solution.

## Conclusion

In this paper, we have addressed the problem of partition allocation in mixed-criticality systems when the goal is to reduce energy consumption. Instead of focusing on new scheduling algorithms to adjust frequency in order to save energy, we propose a partition to CPU allocation that takes into account not only the different frequencies at which the CPU can operate but the level of criticality of the partitions. We have also proposed a different mixed-criticality model instead of the well-known Vestal model. The motivation is to cope with the requirements imposed by the applications used in the avionics and railway sector, since the results of this research will be applied in H2020 project Safepower with demonstrators in these two sectors. We have proposed an allocation method for real-time systems of the same criticality and extended this method for mixed-criticality systems. The extension is based on combining two strategies: dropping partitions that are not mandatory in extreme low power situations and reducing the bandwidth of mandatory LO partitions. In the general method we achieve an energy saving up to a 5%. In the extension to MCS, we achieve up to a 35% saving at the expense of losing performance of LO partitions. We propose a set of profiles so at run time the system has to decide to switch to a more energy conserving profile depending on the power sensor values. In spite of the fact that this is not an exact method, it provides a faster feasible solution, with similar results to the optimal solution.
